# Multiple zoonotic helminth infections in domestic dogs in a rural area of Khuzestan Province in Iran

**DOI:** 10.1186/s12917-018-1529-6

**Published:** 2018-07-25

**Authors:** Molouk Beiromvand, Abdollah Rafiei, Elham Razmjou, Sharif Maraghi

**Affiliations:** 1 Infectious and Tropical Diseases Research Center, Health Research Institute, Ahvaz Jundishapur University of Medical Sciences, Ahvaz, Iran; 2 Department of Parasitology, School of Medicine, Ahvaz Jundishapur University of Medical Sciences, Ahvaz, Iran; 3 Department of Parasitology and Mycology, School of Medicine, Iran University of Medical Sciences, Tehran, Iran; 4 Institute of Health Research, Thalassemia and Hemoglobinopathy Research Center, Ahvaz Jundishapur University of Medical Sciences, Ahvaz, Iran

**Keywords:** *Echinococcus* spp*.*, *Taenia* spp., *Toxocara canis*, *Toxascaris leonina*, *Spirometra erinaceieuropaei voucher*, *Mesocestoides corti.*, Dog, Iran

## Abstract

**Background:**

Echinococcosis and toxocarosis caused by the genus of *Echinococcus* and *Toxocara* spp. are among important helminthic diseases worldwide. Limited data on the prevalence of these parasites persuaded us to determine the prevalence of *E. granulosus, E*. *multilocularis*, and *T. canis* infections in domestic dogs in rural areas of Ahvaz, southwestern Iran. Fecal samples from 167 domestic dogs were examined using both microscopy and PCR techniques. Multiplex PCR was performed for the presence of *Echinococcus*, and *Taenia* spp. and single PCR for detection of *T. canis* and *Toxascaris leonina*.

**Results:**

The total occurrence of identified parasites was 65 (38.9%). The microscopic examinations showed that 40 (24%), 18 (10.8%), and four (2.4%) of dogs were infected with taeniid-like, ascarid, and both genera eggs, respectively. *Echinococcus granulosus* was identified in seven (4.2%), *Taenia* spp. in 29 (17.4%), and mixed infection with both in 11 (6.6%) samples. Sequencing of PCR-positive samples identified *E. granulosus* s.s. (G1), 18 *T. hydatigena* (10.8%), five *T. multiceps* (3%), three *T. serialis* (1.8%), one *T. ovis* (0.6%), one *Spirometra erinaceieuropaei voucher* (0.6%), and two *Mesocestoides corti* (1.2%). This is the first report of *S. erinaceieuropaei voucher* and *M. corti* in dogs in Iran. Nine (5.4%) and 16 (9.6%) dogs showed infection with *T. canis* and *T. leonina*, respectively. Two samples showed coinfection with both ascarids.

**Conclusions:**

Several studies have reported echinococcosis and toxocarosis in intermediate hosts from the southwest of Iran; however, this study is the first molecular research on *E. granulosus* and *T. canis* in domestic dogs in a rural area of southwestern Iran. Furthermore, issues of soil contamination with dogs’ feces and recent dust storms in Khuzestan may have a role in the spreading of these zoonotic infections to other provinces close to it, and neighboring countries such as Iraq.

## Background

The domestic dog as final host of zoonotic enteric parasites plays an important role in the transmission of diseases such as echinococcosis and toxocarosis to humans [1]. Infected dogs can contaminate the environment with excreted eggs, posing a serious public health problem [2]. Toxocarosis, an important helminthic disease, is caused by the larval stages of the nematodes *Toxocara canis* and *Toxocara cati*, the adult forms of which reside in the small intestine of canine and feline hosts, respectively [3]. The worldwide occurrence of *T. canis* in dogs is reported to range from 3.1 to 82.6% [4–7]. Humans can acquire the infection through the ingestion of embryonated eggs via geophagia, contaminated food, and raw vegetables or by ingestion of undercooked meat of infected paratenic hosts [1, 8]. Echinococcosis, an important zoonotic disease with worldwide distribution, is caused by the metacestode stage of tapeworms belonging to *Echinococcus* spp. [9]. Recently, the disease has been included in a subgroup of 17 neglected tropical diseases by the World Health Organization [10]. Cystic echinococcosis (CE) and alveolar echinococcosis (AE), caused by *E. granulosus* and *E. multilocularis*, respectively, have particular medical, economic, veterinary, and public health importance [9].

The life cycle of these cestodes involves dogs and other canids as definitive hosts with domestic and wild ungulates as intermediate hosts of *E. granulosus* and small mammals as intermediate host of *E. multilocularis*. Humans can act as an accidental intermediate hosts through ingestion of *Echinococcus* eggs, which are excreted into the environment in feces of final hosts. Based on molecular studies using mitochondrial genes, 10 strains (genotypes G1–G10) have been recognized, including *E. granulosus* sensu stricto (s.s.) (genotypes G1–G3), *Echinococcus equinus* (the ‘horse strain’, genotype G4), *Echinococcus ortleppi* (the ‘cattle strain’, genotype G5), and *Echinococcus canadensis* (G6–G10) [11, 12]. The genotype G1 is the most common genotype on all continents and the primary strain infecting humans [13]. More than 270 million people living in the Central Asian Republics, Afghanistan, Pakistan, China, and parts of Iran, particularly rural inhabitants, principally farmers and herdsmen, are at risk of CE [14]. Cystic echinococcosis is also one of the most frequently reported zoonoses and an important public health problem in the Middle East [15, 16].

Iran is an endemic focus of CE and AE [16, 17]. Hospital records indicate that approximately 1% of patients seen in surgical wards in Iran are infected with *E. granulosus* [16]. The incidence of human CE in Iran is estimated to be 1.18–3 per 100,000 population [18]. An *E. granulosus* infection prevalence of 5–49% in stray dogs, 88% in sheep, 70% in camels, and 19% in cattle has been recorded in Iran [16]. *Echinococcus granulosus* s.s. (G1–G3) and the camel strain (G6) are causative agents of canine and human echinococcosis in Iran [13, 19], and, in some parts of the country, the G1 strain is the cause of 75–100% of canine infections [20]. *Echinococcus multilocularis* infection was first reported in 1970 in the northwest of Iran [21, 22], and the latest survey in northeastern country revealed *E. multilocularis* infection in domestic and wild canids [17]. Although southwestern Iran is not an endemic area of AE, a human case of AE was reported for the first time in 2012 [23].

In many rural areas of Iran, large populations of dogs are kept for being shepherd dogs and guard dogs, and the prevalence of *E. granulosus* infection in up to 64% in dogs play key roles in the transmission of echinococcosis to humans [14, 24]. A purgation study using arecoline hydrobromide in 13 provinces indicated that 20% of farm dogs in Khuzestan Province were infected with *E. granulosus* [24]. In Khuzestan villages, increasing the dog population, which roam gardens and streets and the lack of hygienic disposal of dog’s feces from the ground lead to soil contamination with intestinal parasites [24, 25]. Moreover, the province had been hit by dust storms many times in recent years [26], which may increase the risk of human infections to soil transmitted pathogens in the rural inhabitants. In order to control the parasitic diseases in this region, new data on the existence of parasitic agents, their prevalence, and transmission routes are considered necessary. Thus, this study was performed using the flotation/sieve technique, microscopy and PCR to determine the prevalence of *E. granulosus, E. multilocularis,* and *T. canis* and *Toxascaris leonina* infections in order to identify the role of domestic dogs in the environmental contamination and transmission of the infections in rural areas of Ahvaz, southwestern Iran.

## Methods

### Study area

Khuzestan Province, one of the 31 provinces of Iran, located in the southwest area of the country near the borders with Iraq and the Persian Gulf, has an area of 63,238 km^2^ and a population of 4,531,720 [27]. The landscape comprises rolling hills and mountainous regions in the north with plains and marshlands to the south. The capital, Ahvaz, (31° 19′ N 48° 40′ E) has a population of 1,112,021 [27]. Three of the country’s largest rivers, the Karoun, Karkheh, and Maroun, make the Khuzestan plain especially suited for agriculture. The climate is hot and occasionally humid. The annual mean of maximum and minimum temperatures is about 50 °C and 9 °C in months of July and March, respectively. The annual precipitation is 150–256 mm in the south and 995–1100 mm in the north [26].

### Sampling

The feces of 167 domestic dogs were collected from rural areas of Ahvaz County, the capital of Khuzestan Province in 2013 and 2014. Simple random sampling was carried out according to information from the local veterinary offices. All samples were placed in zippered plastic bags, labeled with an assigned number, location, and date of sampling and immediately taken to the Parasitology Department, Ahvaz Jundishapur University of Medical Sciences, Iran. For safety and to kill oncospheres of *Echinococcus* spp. eggs [28], all samples were frozen at − 80 °C for at least 7 days, and then held at − 20 °C until examination.

### Egg isolation by flotation/sieving

Taeniid-like and ascarid eggs were isolated from feces by flotation with a zinc chloride solution (density, 1.45 g/ml^− 1^). Approximately 5 g of fecal sample were suspended in 50 ml tap water and mixed until completely dispersed. The fecal suspensions were filtered through 100 μm mesh to remove large particles, and, subsequently centrifuged at 1000 g for 5 min. After discarding the supernatant 12 ml saturated zinc chloride solution was added to fecal sediment and carefully vortexed. Afterwards, the suspension was centrifuged at 1000 g for 30 min, and then sequential sieving through 40 and 21 μm mesh was used for isolating ascarid and taeniid eggs, respectively as described by Mathis et al. [29]. Identification of taeniid and ascarid eggs was performed by light microscopy and then the rest of the sediments (approximately 200 μl) were transferred to 2 ml tubes and stored at − 20 °C until examination.

### DNA extraction

DNA isolation of all samples was performed using the QIAamp DNA Mini Kit (QIAGEN, Germany) according to the manufacturer’s instructions with slight modification of the final step for both taeniid-like and ascarid eggs [17, 30] and increase of incubation time at 56 °C to at least 16 h for ascarid eggs until eggs were completely lysed [31]. Before extraction, five cycles of freezing in liquid nitrogen 5 min and boiling at 100 °C 7 min were conducted to disrupt the parasite egg wall. Duration of the final step was increased to 5 min to improve the yield of DNA [17]. DNA extracts were stored at − 20 °C until PCR was conducted.

### PCR

Multiplex PCR was conducted on all samples for the amplification of the 395 bp fragment of NADH dehydrogenase subunit 1 (*nad1*) gene, in order to detect *E. multilocularis* using the primer pairs Cest1 (5′-TGCTGATTTGTTAAAGTTAGTGATC-3′) and Cest2 (5′-CATAAATCAATGGAAACAACAACAAG-3′), and 117 bp of the small subunit of ribosomal RNA (rrnS) gene to identify *E. granulosus* using the primers Cest4 (5′-GTTTTTGTGTGTTACATTAATAAGGGTG-3′) and Cest5 (5′-GCGGTGTGTACMTGAGCTAAAC-3′), and also *Taenia* spp. to identify 267 bp using the primer sets Cest3 (5′-YGAYTCTTTTTAGGGGAAGGTGTG-3′) and Cest5 (5′-GCGGTGTGTACMTGAGCTAAAC-3′) in conditions as described previously [32]. The PCR reaction was performed in a 25 μl volume reaction using 12.5 μl of the master mix (QIAGEN Multiplex PCR, Germany), 8 μl H_2_O, 2 μl DNA template, and 2.5 μl of primers (2 μM of primers Cest1, Cest2, Cest3, and Cest4 and 16 μM of primer Cest5 in H_2_O) [32].

PCR amplification was performed targeting the internal transcribed spacer 2 (ITS-2) regions of the ribosomal DNA genes to amplify 380 and 300 bp fragments for the identification of *T. canis* and *T. leonina*, using the forward primers *Tcan*1 (5′-AGTATGATGGGCGCGCCAAT-3′) and *Tleo*1 (5′-CGAACGCTCATATAACGGCATACTC-3′), respectively and reverse primer NC2 (5′-TTAGTTTCTTTTCCTCCGCT-3′) on all fecal samples [33]. Amplifications were carried out on a 25 μl reaction volume containing 12.5 μl of the master mix (Qiagen, Hilden, Germany), 9 μl distilled water, 2.5 μl DNA template, and 0.5 μl of each primer under the following described protocol with slight modifications: An initial denaturation step at 95 °C for 5 min; 35 cycles of 94 °C for 20 s, 58 °C for 30 s, and 72 °C for 30 s; and a final extension cycle at 72 °C for 10 min [33]. For each amplification, one negative control (distilled water) as well as one positive control contains DNA of *E. multilocularis*, *E. granulosus*, *Taenia hydatigena*, *T. canis*, and *T. leonina* sample was included.

PCR amplicons of 395, 267, and 117 bp from *E. multilocularis*, *Taenia* spp., and *E. granulosus* were analyzed by 2% agarose gel electrophoresis, and the 380 and 300 bp fragments from *T. canis* and *T. leonina* were electrophoresed on 1.5% agarose gel stained with ethidium bromide, and visualized with a chemiluminescence imaging system (Syngene, Cambridge, UK).

### Sequencing

For species identification of amplified 267 and 117 bp fragments, PCR-positive samples were subjected to single PCR using Cest3 and Cest5 primer pairs for *Taenia* spp. and Cest4 and Cest5 primer pairs for *E. granulosus*. PCR products were purified using the MinElute PCR purification Kit (Qiagen, Germany) according to the manufacturer’s instructions and directly sequenced at the Bioneer Co. (Daejeon, South Korea) with the primers Cest4 and Cest5_seq_ for *E. granulosus s.s.* and *Taenia* spp., respectively.

### Statistical analyses

The frequency of parasite occurrence in the samples and respective 95% confidence intervals (CI) were calculated using SPSS 18 software (SPSS Inc., Chicago, IL, USA).

## Results

### Microscopic analysis

Of the 167 fecal samples examined by light microscopy, 65 (38.9%; 95% CI: 32–46) samples were positive for ascarid and taeniid-like eggs. Of these, 43 (25.7%; 95% CI: 20–33) were identified as taeniid-like, 18 (10.8%; 95% CI: 7–16) as ascarid, and four (2.4%; 95% CI: 1–6) showed mixed infection with taeniid-like and ascarid eggs.

### PCR analysis

**Fig. 1:**
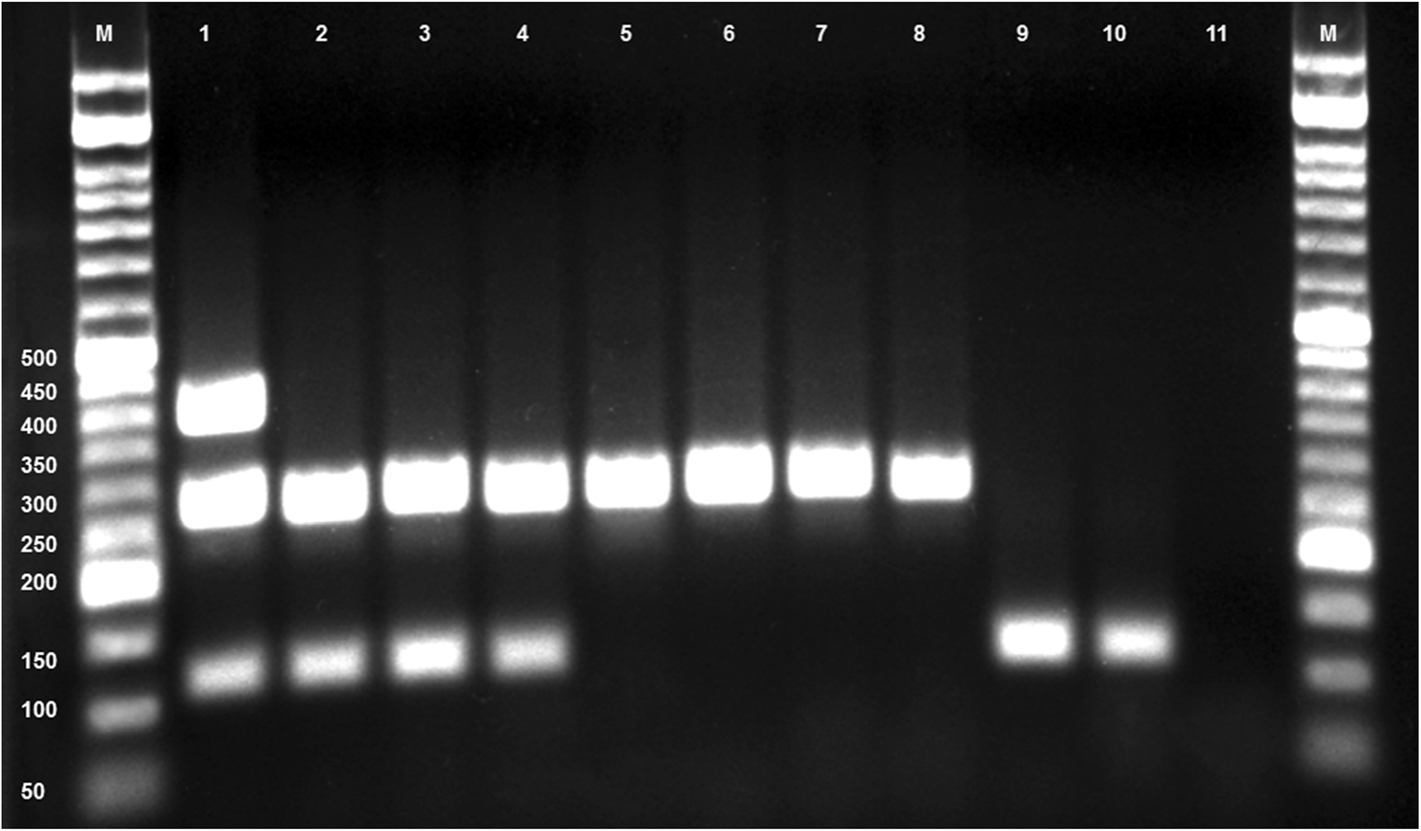
Multiplex PCR amplification of mitochondrial genes of *Echinococcus granulosus* and *Taenia* spp*.* from eggs in feces of dogs from Khuzestan Province, Iran. Lane M, 50 bp DNA ladder; Lane 1, positive control, a mixture of standard DNA of *E. multilocularis* (395 bp), *E. granulosus* (117 bp), and *T. hydatigena* (267 bp); Lane 2–10, positive samples; Lane 11 negative control

The multiplex PCR revealed mono-infections by *E. granulosus* in seven (4.2%; 95% CI: 2–8) and *Taenia* spp. in 27 (17.4%; 95% CI: 11–23) while 13 (6.6%; 95% CI: 5–13) showed mixed infection by *E. granulosus, Taenia* spp., *T. canis*, and *T. leonina*. *Echinococcus multilocularis* infection was not identified in any sample (Fig. 1).

Of the 167 samples, 22 (13.2%; 95% CI: 9–19) were identified as ascarid by light microscopy. However, PCR results showed that 25 samples were infected with ascarid. Of these, *T. canis* was detected in nine (5.4%; 95% CI: 3–10) and *T. leonina* in 16 (9.6%; 95% CI: 6–15) samples. Two samples (1.2%; 95% CI: 0–4) showed co-infection by both species.

Based on PCR results, fifty (29.9%) dogs showed mono-infection, and 15 (9%) had mixed infections, including 12 (7.2%; 95% CI: 4–12) infected with two, and three (1.8%; 95% CI: 1–5) infected with three, genera of the identified parasites. *Taenia* spp. were the most frequently detected parasites (24%), with *E. granulosus*, found in 18 investigated dogs (10.8%), the second most common (Table 1).

**Table 1:** Number and frequency (%) of single and co-infections with *Echinococcus granulosus*, *Taenia* spp., *Toxocara canis*, and *Toxascaris leonina* in fecal samples by PCR

Parasite species	N (%)	95% CI
*E. granulosus*	7 (4.2%)	2–8
*Taenia* spp.	27 (16.2%)	11–23
*T. canis*	5 (3%)	1–7
*T. leonina*	11 (6.6%)	4–11
*E. granulosus*, *Taenia* spp.	9 (5.4%)	3–10
*Taenia* spp., *T. leonina*	1 (0.6%)	0–3
*T. canis*, *T. leonina*	2 (1.2%)	0–4
*E. granulosus*, *Taenia* spp., *T. canis*	1 (0.6%)	0–3
*E. granulosus*, *Taenia* spp., *T. leonina*	1 (0.6%)	0–3
*Taenia* spp., *T. leonina*, *T. canis*	1 (0.6%)	0–3

### DNA sequences analysis

The obtained sequences were edited and aligned in MEGA 6.0 using ClustalW and compared to published GenBank sequences of *E. granulosus s.s.* (G1) (AF297617), *E. granulosus s.s.* (G3) (KJ559023), *Taenia hydatigena* (GQ228819), *Taenia multiceps* (GQ228818), *Taenia ovis* (AB731675), and *Taenia serialis* (AB731674) by BLAST.

Ten isolates with sufficient DNA were sequenced. The obtained sequences of the *rrnS* gene of *E. granulosus* revealed the presence of *E. granulosus s.s.* (G1 genotype) in all sequenced samples.

The sequences from 30 *Taenia* spp. isolates revealed *T. hydatigena* in 18 (10.8%), *T. multiceps* in five (3%), *T. serialis* in three (1.8%) dogs, and *T. ovis* in one (0.6%) dog. The Cest3/Cest5 primers are not specific for *Taenia* spp., thus they can detect non-taeniid cestodes such as *Mesocestoides* spp., *Dipylidium caninum* and *Diphyllobothrium latum* [32]. For this reason, the sequence of one sample identified *Spirometra erinaceieuropaei voucher*, and DNA sequencing of the remaining two amplicons was positive for *Mesocestoides corti* (Table 2). All isolates showed 99% or greater identity with sequences in the GenBank database. Our sequences were submitted to GenBank with the accession numbers listed in Table 2.

**Table 2:** Number of infections and frequency (%) of *Echinococcus granulosus, Taenia* spp., *Spirometra erinaceieuropaei voucher*, and *Mesocestoides corti* isolated from dog feces

Species	N (%)	95% CI	Accession numbers
*Taenia multiceps*	5 (3)	1–7	LC111533–LC111537
*Taenia serialis*	3 (1.8)	1–5	LC113990–LC113992
*Taenia ovis*	1 (0.6)	0–3	LC113989
*Echinococcus granulosus* s.s. (G1)	10 (6)	3–11	LC102348, LC114210–LC114215 LC114273–LC114275
*Taenia hydatigena*	18 (10.8)	7–16	LC106307–LC106308, LC106356–LC106360, LC107625–LC107626 LC107472–LC107474, LC107781–LC107783, LC109311–LC109313
*Spirometra erinaceieuropaei voucher*	1 (0.6)	0–3	–
*Mesocestoides corti*	2 (1.2)	0–4	–

## Discussion

The obtained results provide the first molecular evidence of *E. granulosus* genotype G1 in dogs from the rural areas of Khuzestan Province, southwestern Iran. The previous studies showed that sheep and camel strains (G1 and G6) are the main cases of CE in Iran [13, 19, 34], in which G1 is the most common and the primary causative agent of the majority of canine and human infection [20, 35]. Our data are in agreement with the earlier studies that demonstrate the dominant genotypes in canids are G1–G3 genotypes [16, 20, 34]. The finding prevalence of 10.8% for *E. granulosus* are along with studies carried out in other parts of Iran that have shown a prevalence of 7.4% in the southeast [36], 13.2% in the west [37], 16.9% in the northeast [17], 20% in the northwest [38], and 64% in the central part of the country [39]. Eslami et al. reported that 20% of farm dogs was infected with *E. granulosus* in the only study conducted in the study area. It is possible that the lower prevalence in this survey compared to the previous study is due to factors such as the studied population. In the study conducted by Eslami et al., only farm dogs that spent more time roaming were investigated; therefore, they always had easy access to discarded offal, which resulted in a higher chance of infection [24]. In addition, it should be considered that the rate of infection has decreased during the recent years in all over the country in human and dogs. This phenomenon may be results of a growing awareness of people and improving the quality of healthcare in Iran. High reported prevalence of infection in the northwest [38], and central part of the country [39] might be related to geographical locations and environmental temperature. Khuzestan Province has dry and hot weather, and in summer the temperature can rise to over 50 °C in some regions, including Ahvaz [40], so it may decrease the survival of eggs in summer. Under optimal conditions such as high precipitation, moist soil, and low temperature, taeniid and ascarid eggs can survive at least for one year, which provide opportunities for human infections [41, 42]. Despite the fact that climatic conditions in Khuzestan Province in summer may decrease the possibility of the infection, the role of domestic dogs in environmental contamination and human infection should not be ignored.

Rural inhabitants comprise approximately 15% of the over 4.5 million people living in Khuzestan Province [27]. In many rural homes, large populations of dogs are kept for being shepherd and guard dogs, so the *E. granulosus*-infected dogs play key roles in the transmission of echinococcosis to humans through contamination of soil with its feces as mentioned in previous studies [14, 24]. In recent years, because of El Niño Southern Oscillation (ENSO), dust storms have affected Iran and other Persian Gulf countries. Based on the reported data, one of the main targets of dust storms is the southwest of Iran [43]. Given that the streets of Khuzestan villages are muddy and unpaved, these can lead to the spread of parasitic agents and the contamination of human environment, food, vegetables, and, particularly, water supplies of rural inhabitants. Furthermore, it is a serious threat to other provinces near to Khuzestan.

Despite a report of human AE from Khuzestan Province [23], its causative agent, *E. multilocularis*, was not identified in the dogs examined. Therefore, it seems that the reported case [23] should be infected in a place other than Khuzestan with more suitable environment conditions for *E.* multilocularis life cycle. Although, with respect to the lower prevalence of *E. multilocularis* infection among domestic dogs compared to other definitive hosts such as red fox [17, 44], for reaching to the stringent deduction a widespread investigation covering more samples and areas is essential.

The occurrence of *T. hydatigena*, *T. multiceps*, and *T. ovis* in investigated dogs indicates the potential of domestic dogs as definitive hosts that may acquire infection by scavenging on infected domestic herbivorous mammals, principally sheep carcasses. Lethal infection with some *Taenia* spp., particularly *T. multiceps*, in intermediate hosts such as sheep, is significant, causing considerable economic losses [45, 46]. These species have also been reported to have zoonotic importance, with several human cases recorded worldwide [47]. *Taenia hydatigena* (53%) and *T. multiceps* (3–40%) are among the most prevalent *Taenia* spp. in dogs in Iran [45, 48], with the economic losses due to *T. multiceps* infection in sheep estimated at 15,700 USD in 1990–1992 [49]. Despite economic importance caused by *T. hydatigena* and *T. multiceps* in intermediate hosts, no molecular epidemiological data were recorded in definitive hosts from southwestern Iran. Furthermore, molecular examinations confirmed the presence of *S. erinaceieuropaei voucher* infection in one dog*.* According to our knowledge, this is the first report of *S. erinaceieuropaei voucher* in Iranian dogs. *Spirometra erinaceieuropaei,* is one of the most important cestodes, causes sparganosis in human [50]. Human infection occurs mainly through consumption of raw or undercooked meat of infected animals with plerocercoids, such as snakes and frogs, water contaminated with cyclops, or by using frog or snake flesh in traditional medical practices [51]. Moreover, the existence of *M. corti* (syn. *M. vogae*) in two dogs confirmed by sequencing results. This tapeworm is mainly a mice parasite but is also a human life-threatening cestodes similar to *E. multilucularis* and *Taenia solium* [52]. Although, another species of this genus, *M. lineatus*, has been reported from stray dogs (26.5%) in the west of Iran [37], this is the first time that *M. corti* reported in dogs of Iran.

Other important zoonotic helminthes, *T. canis* (5.4%) and *T. leonina* (9.6%), was reported in the present study. There is a dramatically difference in the reported prevalence of *T. canis* infection in domestic and stray dogs from 60% in the north [39] and 22% in the central [6] to 6% in the western [37], 4.3% in southeast [53] and 5.4% in southwestern (this study) of Iran. It seems that the observed differences in the investigated areas is dependent on different factors such as the geographical location, temperature and humidity, soil type, vegetation cover, or the age of the examined dogs [41, 54].

## Conclusions

This study has demonstrated relatively high prevalence of zoonotic parasites (38.9%) and the first reports of *S. erinaceieuropaei* and *M. corti* in domestic dogs from Khuzestan Province. Despite the lower prevalence of *E. granulosus* and *T. canis* compare to other parts of the country, the large population of dogs, particularly stray dogs are significant. Therefore, these findings are important in two respects: the first, the obtained data indicate a serious risk for echinococcosis and toxocarosis, the latter, these findings reflect a possible role of dogs in transmission of human sparganosis in the area. Despite the fact that in Khuzestan Province people avoid traditional medical practices by using snake or frog flesh, the risk of transmission by contaminated water should not be ignored. Thus, more investigations on *S. erinaceieuropaei* and *M. corti* among Khuzestan rural dogs is essential. Moreover, control and preventive programs, including health education, anthelminthic treatment of dogs, and supervision of home slaughtering, should be implemented to reduce the transmission of zoonotic parasites to the inhabitants of villages.

## Abbreviations

°C: Centigrade
AE: Alveolar echinococcosis
BLAST: Basic Local Alignment Search Tool
bp: Base pair
CE: Cystic echinococcosis
Cest: Cestodes
CI: Confidence Interval
DNA: Deoxyribonucleic acid
E: East
*E. granulosus*: *Echinococcus granulosus*
*E*. *multilocularis*: *Echinococcus multilocularis*
*Echinococcus granulosus* s.s.: *Echinococcus granulosus* sensu stricto
ENSO: El Niño Southern Oscillation
G: Genotype
g: Gram
g: Gravity
h: Hour
ITS: Internal transcribed spacer
km^2^: Square kilometre
*M. corti*: *Mesocestoides corti*
*M. lineatus*: *Mesocestoides lineatus*
min: Minute
ml: Milliliter
mm: Millimeter
N: North
*nad1*: NADH dehydrogenase subunit 1
PCR: Polymerase chain reaction
rrnS: Small subunit of ribosomal RNA
*S. erinaceieuropaei voucher*: *Spirometra erinaceieuropaei voucher*
sec: Second
seq: Sequencing
spp.: The plural form of species
SPSS: Statistical Package for the Social Sciences
*T. canis*: *Toxocara canis*
*T. hydatigena*: *Taenia hydatigena*
*T. leonina*: *Toxascaris leonina*
*T. multiceps*: *Taenia multiceps*
*T. ovis*: *Taenia ovis*
*T. serialis*: *Taenia serialis*
*Tcan*: *T canis*
*Tleo*: *T leonina*
UK: United Kingdom
USD: United States Dollar
μl: Microliter
μm: Micrometer
μM: Micromolar
